# Investigating the perception of Romanian adults on ophthalmology services from an experiential marketing perspective

**DOI:** 10.22336/rjo.2017.33

**Published:** 2017

**Authors:** Consuela-Mădălina Gheorghe, Iuliana-Raluca Gheorghe, Victor Lorin Purcărea

**Affiliations:** * Department of Marketing and Medical Technology, “Carol Davila” University of Medicine and Pharmacy, Bucharest, Romania

**Keywords:** perception, ophthalmology services, experiential marketing, snowball sampling technique, ophthalmology sector

## Abstract

Nowadays, we live in a world in which we are daily bombed by hundreds of advertisements. Specialists have to discover other channels or embed attractive elements in the advertisements’ messages to cut through the clutter and catch the consumers’ attention. The evolution of the concept of service has changed from the commercial status to determining a lifestyle.

Buying a service has led to a change in the consumer behavior. Consumers want to buy services that dazzle their senses, touch their hearts, and stimulate their minds, not as before, excellent or satisfying.

Ophthalmology is the medical specialty that is the most oriented toward outpatient care, as hospitalization is required only in a small percentage of cases.

The objective of this case study was to investigate the perception of Romanian adult consumers on ophthalmology services from an experiential marketing perspective, by using the Focus Group method.

Ophthalmology requires a wide range of skills due to the diversity of consumers who demand specialized consultations.

Experiential marketing is a valuable strategy that ophthalmologic organizations may use to target specific consumers.

The purpose of this case study was to identify the perceptions of Romanian adults on experiential marketing campaigns and determine the degree to which these campaigns influenced their decisions of buying an ophthalmologic service.

Using a snowball sampling technique we have sent a filter questionnaire to 40 people on the internet. The filter questionnaire consisted of questions about wearing eyeglasses, the period of wearing them, the last ophthalmologic consultation, the type of ophthalmologic clinic they were going to, age and education.

The respondents revealed there is almost no visibility on promoting services even if there is an upsurge of organizations offering this type of health service in the Romanian ophthalmology sector.

## 1.Introduction

Nowadays, we live in a world in which we are daily bombed by hundreds of advertisements. Moreover, as consumers found new methods of avoiding advertisements, the impact of sending the ad’s message on traditional channels has diminished. Consequently, specialists have to discover other channels or embed attractive elements in the advertisements’ messages to cut through the clutter and catch the consumers’ attention. 

Further, the evolution of the concept of service has changed from the commercial status to determining a lifestyle [**1**]. More exactly, buying a service has led to a change in the consumer behavior, as they became more educated, experienced, sophisticated, demanding and discerning in their purchasing process, which developed into greater requirements for the marketing experts. At the same time, consumers have started to consider the purchasing process a relationship established between them and the service itself [**2**]. In addition, consumers want to buy services that dazzle their senses, touch their hearts and stimulate their minds, not as before, excellent or satisfying [**3**]. This is where, more and more often, the interest of marketing experts to provide service experiences with a unique sensation has risen, rather than provide a list of features and benefits that remain engrained in the consumers’ minds for a long period of time, through experiential marketing. 

Health care is a service that *most people need but do not want*, in other words, it is an unwanted service. In addition, health care services are highly troublesome but critically important for a population’s wellbeing [**4**]. From a marketing perspective, it is the most customized service that the consumers can buy; however, its variability in solving health problems stands as proof that there is an undeniable difference between health care services and the other services. For instance, Berry and Beaudapudi (2007) [**4**] pinpointed that consumers are sick, relinquish privacy and are reluctant in disclosing their most intimate thoughts, perceiving a high risk in the service delivery, as it also happens in ophthalmology. 

Ophthalmology is the medical specialty that is the most oriented toward outpatient care, as hospitalization is required only in a small percentage of cases, when surgery is conducted. Further, many ophthalmologic patients have no disease, but require preventive check-ups and eyeglasses for benign refractive conditions, being self-referred or referred non-specifically by other physicians for a specialized consultation. Ophthalmologic practice is like primary care, with a large throughput of patients, many of whom are normal [**5**].

Since ophthalmology services are credence oriented and consumers are self-referred, marketing experts use an experience to attract a target audience with the intention of triggering an emotion or action at a cognitive level [**4**]. The experiential approach is the new currency of the modern marketing landscape, because experiences are life and people talk about life experiences day-by-day [**6**]. 

The objective of this case study was to investigate the perception of Romanian adult consumers on ophthalmology services from an experiential marketing perspective, by using the Focus Group method. 

## 2.Ophthalmology specialty from a marketing perspective 

Ophthalmology requires a wide range of skills due to the diversity of consumers who demand specialized consultations. Thus, patients who need an ophthalmic consultation range from pediatric to geriatric segments. Patient care usually takes place on an outpatient basis on a long period of time, allowing the development of a true report. Moreover, surgeries are usually performed on a day care basis and under local anesthesia. 

According to Thomas (2010), many ophthalmologic consumers are different from other service consumers in some aspects. The main differences between the ophthalmologic consumers are highlighted in **Table 1**. 

**Table 1:** Differences between ophthalmologic and other services consumers

*Ophthalmologic services*	*Other services*
Seldom are the last decision makers	Usually are the ultimate decision makers
Often make decisions subjectively	Usually make decisions objectively
Seldom know the price	Always know the price
Seldom make decisions based on price	Usually make decisions based on price
Usually make nondiscretionary purchases	Usually make discretionary purchases
Usually require a professional referral	Rarely require a professional referral
Have limited knowledge of service attributes	Have significant knowledge of service attributes
Have limited ability to judge the quality of the service	Are usually able to judge the quality of the service
Have limited ability to evaluate the outcome	Are usually able to evaluate the outcome
Are not susceptible to standard marketing techniques	Are susceptible to standard marketing techniques
Source: adapted from Thomas RK. Marketing Health Care Services. 2010, Oxford, Oxford University Press, 157.	

## 3.Experiential marketing in ophthalmologic services 

Experiential marketing is a valuable strategy that ophthalmologic organizations may use to target specific consumers, which delivers a compelling service experience that is appealing to the wants and needs of its consumers. Moreover, experiential marketing connects with consumers through the participation and tangible nature of a personally relevant and memorable experience [**7**]. 

The concept of experiential marketing was first introduced in 1999 by Bernd Schmitt, who defined it as the consumer’s process of recognition or intention to purchase a service only after they have had an experience with the organization, through which the value perceived by the consumer has increased. Nevertheless, experiential marketing does not refer to the quality, the benefits, and the functions of the service, but is rather emotionally oriented as it conveys to the consumer and the senses it activates. 

The core values of emotional marketing are the following [**8**]:

1. Communicate by using the sense of touch and emotions more while helping consumers visualize the way the service will bring a benefit to their lives. 

2. Demonstrate the use and benefit by employing photography and other illustration techniques, showing the application, use and service benefits in an “aspirational” manner. 

3. Infuse emotional selling messages, as the consumers’ buying decisions are more emotionally oriented than logic. 

By relying on the strategic experiential model, Schmitt divided the types of marketing into 5 categories: Sense Experience, Feel Experience, Think Experience, Act Experience, and Relate Experience [**9**].

- **Sense Experience** refers to the experience consumers gain while using their senses such as sight, hear, touch, smell and taste. As such, through sensory experience, consumers will be able to develop experience logic and, subsequently, using the experience logic, they will form attitudes to differentiate services [**10**]. 

In fact, Sense Experience appeals to the five basic human senses, as it follows [**11**]:

a. Visual Experience. Sight is the most commonly used sense in marketing because it is the most stimulated by the environment. Due to this fact, the ophthalmologic organizations use many colors and forms to attract consumers, because they are memorized easier in the audience’ mind. Moreover, the colors of the surroundings, lights, materials, or layout are also important criteria to take into consideration in selling points.

b. Auditory experience uses music or sounds to promote a message in order for the consumer to remember it easier. 

c. Tactile experience. 

d. Olfactory experience is connected to the emotions and ophthalmologic organizations use natural or artificial smells. 

- **Feel Experience** is referred to the consumer’s inner emotion, mood and feeling, which is derived from the consumption experience [**12**]. Feel experience can only work if there is a deep understanding of which stimuli trigger certain emotions. 

- **Think Experience** refers to the stimulation of the consumer’s creativity on a cognitive level so that they engage in solving real problems. Moreover, think experience uses surprise, intrigue, and provocation to appeal to problem solving capabilities. 

- **Act Experience** involves the consumer’s activities related to changing a certain behavioral habit or lifestyle. These experiences sometimes occur in a private environment, but are most often the result of interactions between several persons. According to Schmitt, act experience enables consumers to develop experiences that deal with the consumer’s physical body, behavior and lifestyle and the experience gained from the social interaction with other people. Through the experience action, consumers develop a sense of sensation, influence, and relationship with the services offered. 

- **Relate Experience** refers to an experience that allows the consumer to establish ties with various entities and communities through the process of buying and consuming. Relate experience allows consumers to build their connection with the social communities and social entities through the process of purchasing and service consumption [**13**]. 

The ultimate strategy of experiential marketing is to build holistic experiences with intense, broad, and interconnected experiences by using advertising.

**Fig. 1:**
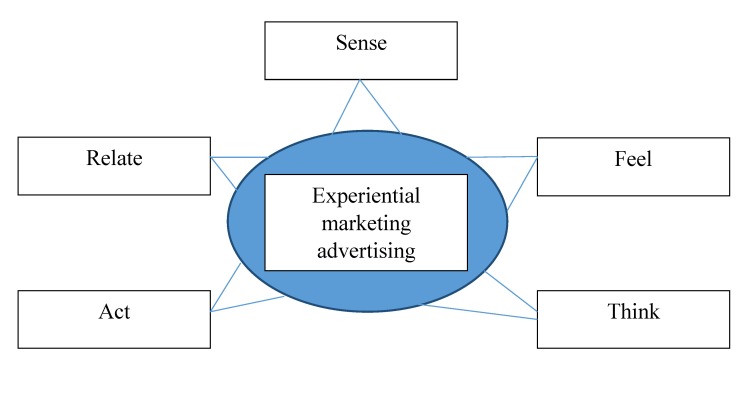
Experiential marketing advertising and the 5 senses

## 4. Research objectives 

The purpose of this case study was to identify the perceptions of Romanian adults on experiential marketing campaigns and determine the degree to which these campaigns influenced their decisions of buying an ophthalmologic service. The objectives of the research were the following:

1. Determine the respondents’ degree of knowledge on the concept of experiential marketing. 

2. Determine the respondents’ degree of knowledge of the concept of experiential marketing applied in ophthalmologic services. 

3. Identify the respondents’ perception of experiential marketing in ophthalmology.

4. Examine the factors that made experiential marketing efficient in ophthalmologic services. 

5. Investigate whether the respondents would go to a consultation in an ophthalmologic clinic advertised with the help of experiential marketing messages. 

## 5. Research methodology 

The focus group method was used in order to assess the established objectives. The focus group is a method specifically used in exploratory research. Moreover, focus groups are small groups of people brought together with the aim of gaining information on a relevant topic, such as generating ideas, learning the respondents’ vocabulary when relating to a certain type of service, or gaining some insight into basic needs and attitudes [**14**]. On one hand, the main advantages of focus groups are that they generate fresh ideas, allow moderators to observe their respondents, may be directed towards understanding a wide variety of issues and allow a fairly easy access to special respondent groups, and, on the other hand, the major disadvantages of focus groups are that they are not representative samples, it is difficult to interpret the results of focus groups, as the moderator’s report is based on a subjective evaluation of what was said during the focus group, and the cost per participant is high [**14**]. 

We decided that 25 people would give us enough insight to assess the objectives. Using a snowball sampling technique we have sent a filter questionnaire to 40 people on the internet. The filter questionnaire consisted of questions about wearing eyeglasses, the period of wearing them, the last ophthalmologic consultation, the type of ophthalmologic clinic they were going to, age and education. We selected only the persons who wore eyeglasses for a period longer than 5 years, who went to a consultation not more than 2 months before the research took place in a private clinic, were aged 25 to 35 years and graduated from university. The selected sample size consisted of 20 participants. Consequently, the participants received 2 invitations with dates, hours and the places where the focus groups took place. The location selected was in a room specially designed for these kinds of research methods, in a marketing research institution in Bucharest, which was rented for a couple of hours. The room also required a projector, because the experiential marketing technique consisted of an advertisement of an ophthalmologic clinic, which needed visualization on a screen. The advertisement can be found on the internet, at https://www.youtube.com/watch?v=bE4eiuoMmAE. It embedded elements that covered all 5 experiences described as being part of the experiential marketing, with a focus on the feel, think and act experiences. 

The focus groups took place during 2 consecutive days and lasted not more than 2 hours. Moreover, the answers were recorded with the help of a mobile phone. The first group consisted of 10 participants and, the second group, of only seven, because one respondent quit in the last minute, and others did not show up even if they confirmed their participation. Hence, 17 persons were included in the focus groups. A questionnaire was administered at the end of every focus group, in case there were respondents who did not have the chance to express themselves or were not very talkative. The recorded conversations were transcribed in a text format and a content analysis was conducted. 

## 6. Findings

Both focus groups took place in a relaxed atmosphere and the 17 participants were friendly and brief on the provided answers, eager to share their knowledge and experiences. The demographic profile of the respondents revealed 4 males and 13 females, the mean period of wearing eyeglasses was of 8 years and the mean period since the respondents went to their last ophthalmologic consultation was of about 5 months. 

After watching the advertisement, the moderator presented the main objective of the research to the respondents, assured them that their answers will be confidential, that they were going to be recorded and if they felt embarrassed while answering a question they could avoid it. 

The first discussion subject approached was about the degree of knowledge of the respondents on experiential marketing. 4 participants answered that it was related to experience and others (10 persons) used words such as testing, trying and selling a service with the sense of experience. 3 respondents did not know what experiential marketing meant. Further, the moderator explained what experiential marketing was, as acknowledged in the scientific literature. 

The second subject approached was whether the participants had any knowledge about the application of experiential marketing in ophthalmologic services. All respondents replied they have not seen any advertisement on ophthalmologic services on Romanian TV or over the internet. However, 2 participants have seen an ad for ophthalmologic services during a dedicated event but they did not use experiential marketing elements. The vast majority of respondents (15 persons) agreed on the lack of advertising in health care services, and implicitly, on ophthalmologic services. 

The third subject covered the factors that would make experiential marketing efficient in ophthalmologic services. The vast majority of respondents took into consideration what the moderator explained about the significance of the concept of experiential marketing and pointed out the following particularities of experiential marketing advertising for ophthalmologic services:

- **All respondents agreed** that the advertisement should be visually attractive and tell a story.

- **13 respondents emphasized** the importance of emotions in advertising, gaining more attention if used properly.

- **All respondents mentioned** the word “empathy” in relation to the advertisement watched. What should be added is that empathy presupposes the relate experience, as part of experiential marketing. Moreover, consumers have to be engaged in the process decisions regarding their health and so, organizations have to empower them and offer a benefit to their experience with the provider. 

The fourth subject discussed focused on the respondents’ intention to go to a consultation of an ophthalmologic organization advertised and which used experiential marketing elements. The vast majority of respondents (13 persons) answered that they would go to a consultation if they could watch the advertisement on TV first. However, a few participants were rather skeptical on trusting the organization when making health care decisions and answered they would rather prefer a recommendation from family and peers, or even the general practitioner instead. 

## 7. Discussion

It is acknowledged that marketers use experience to influence a target audience. Regardless of the field, there is always the intention to use an experience to distribute a message or content which trigger an emotion or action (a behavioral response) [**4**]. 

Moreover, when developing a marketing plan, especially an experiential marketing plan, it is important for the organizations to focus on several factors such as the following [**7**]: 

- Choosing an appropriate location or venue where the ad would be distributed;

- Targeting the appropriate audience (Consumer emotional attitudes vary depending on age, gender, culture, socioeconomic status);

- Creating a fun and memorable experience by using multiple media platforms, visual and print ads;

- Offering consumers what they want to see and hear.

Health care is still a sensitive subject and, when advertising their services, most medical organizations concentrate on a description of indoor components of their building and equipment. Thus, most consumers would not consider this kind of advertisement interesting even if it uses some experiential marketing strategies. As our research findings suggested, the advertising message should enclose emotional elements and add value to the consumers’ experiences. If consumers were influenced by the emotional marketing advertisement they would recommend it to other peers, would build on loyalty and stimulate a purchasing behavior. 

The respondents revealed there is almost no visibility on promoting services even if there is an upsurge of organizations offering this type of health service in the Romanian ophthalmology sector. Moreover, organizations should conceive their advertisements around a story that the consumer could identify with. 
